# Predicting Total Knee Replacement from Symptomology and Radiographic Structural Change Using Artificial Neural Networks—Data from the Osteoarthritis Initiative (OAI)

**DOI:** 10.3390/jcm9051298

**Published:** 2020-05-01

**Authors:** Stephan Heisinger, Wolfgang Hitzl, Gerhard M. Hobusch, Reinhard Windhager, Sebastian Cotofana

**Affiliations:** 1 Department of Orthopedics and Trauma Surgery, Medical University of Vienna, 1090 Vienna, Austria; gerhard.hobusch@meduniwien.ac.at (G.M.H.); reinhard.windhager@meduniwien.ac.at (R.W.); 2 Research Office, Biostatistics, Paracelsus Medical University, 5020 Salzburg, Austria; wolfgang.hitzl@pmu.ac.at; 3 Department of Ophthalmology and Optometry, Paracelsus Medical University, 5020 Salzburg, Austria; 4 Research Program Experimental Ophthalmology and Glaucoma Research, Paracelsus Medical University 5020 Salzburg, Austria; 5 Department of Clinical Anatomy, Mayo Clinic College of Medicine and Science, Rochester, MN 55905, USA; cotofana.sebastian@mayo.edu

**Keywords:** Osteoarthritis, Knee, Total Knee Replacement, Neural Networks, Prediction model

## Abstract

The aim of the study was to longitudinally investigate symptomatic and structural factors prior to total knee replacement (TKR) surgery in order to identify influential factors that can predict a patient’s need for TKR surgery. In total, 165 participants (60% females; 64.5 ± 8.4 years; 29.7 ± 4.7 kg/m^2^) receiving a TKR in any of both knees within a four-year period were analyzed. Radiographic change, knee pain, knee function and quality of life were annually assessed prior to the TKR procedure. Self-learning artificial neural networks were applied to identify driving factors for the surgical procedure. Significant worsening of radiographic structural change was observed prior to TKR (*p* ≤ 0.0046), whereas knee symptoms (pain, function, quality of life) worsened significantly only in the year prior to the TKR procedure. By using our prediction model, we were able to predict correctly 80% of the classified individuals to undergo TKR surgery with a positive predictive value of 84% and a negative predictive value of 73%. Our prediction model offers the opportunity to assess a patient’s need for TKR surgery two years in advance based on easily available patient data and could therefore be used in a primary care setting.

## 1. Introduction

Osteoarthritis (OA) contributes strongly to one individual’s global disability and has been shown to be the leading cause of immobility and impaired health related quality of life in the elderly as compared to any other chronic disease [1,2,3,4]. End-stage osteoarthritis of the knee can be understood as a total organ failure of the synovial knee joint resulting from damage and subsequent loss of function of the involved structures: bone, meniscus, synovium, synovial fluid and cartilage [5]. To date, there is still no curative treatment available, and thus, the ultimate cure remains the total surgical replacement of the affected knee joint.

Radiographic evidence of end-stage knee OA and consistent pain refractory to treatment have been postulated to be the leading key indicators for the taking the decision for total knee replacement (TKR) surgery [6,7]. Of those two criteria, the x-ray imaging-based assessment of the knee joint is the more objective and reliable method and has been shown to serve as a good parameter when trying to evaluate the patient’s need for TKR [8,9,10,11,12]. However, as it has been shown that the x-ray status of the knee does not fully reflect one patient’s pain and symptom status, there is a need to include symptom parameters such as pain, loss of function and quality of life and to balance each factor on an individual basis when taking the decision for performing TKR surgery [13].

A previous report from 2003 by Escobar et al. tried to identify factors for the appropriateness of the indication for TKR in patients with osteoarthritis by using a modified Delphi panel judgment based on outcome measures and confirmed that symptomatology and radiology explained most of the variability in the appropriateness of indication [14]. A previous study by Hitzl et al. [15] revealed that individuals receiving a TKR within a period of 4 years displayed a significantly reduced cartilage thickness at base-line when compared to controls, indicating a change in structure; here, the magnetic resonance imaging (MRI) parameter is a predicting factor that can guide a surgeon’s decision for performing TKR surgery.

Various studies have determined the radiographic status based on conventional radiographs and MRI to be predictive for TKR [16,17,18,19]. Other studies, conducted by various authors, focused on knee specific symptomology and demographic factors [18,19,20].

To date, no study analyzed in detail the longitudinal change in radiographic structural change as assessed via Kellgren and Lawrence grades and knee specific symptomology, i.e., pain, function and quality of life in a 4-year period prior to the TKR surgery. Therefore, the aims of the present study were to investigate structural, symptomatic, socio-economic and demographic factors in a four-year period prior to TKR surgery in order to identify which of those factors were of influence to a surgeon’s decision to performing TKR surgery. We additionally performed statistical analyses based on self-learning neural networks to design a model based on the factors described above for predicting a patient’s need for TKR.

## 2. Material and Methods

### 2.1. The Osteoarthritis Initiative

The Osteoarthritis Initiative (OAI) is a National Institutes of Health (NIH) and privately funded longitudinal cohort study of subjects with, or at risk of developing, symptomatic knee OA [10,11]. Data used in the preparation of this article were obtained from the Osteoarthritis Initiative (OAI) database, which is available for public access at https://nda.nih.gov/oai/. Specific datasets used are OAI, version 3.2.1 clinical data, San Francisco, CA, USA [21]. Individuals of multiple ethnicities, aged 45–75 years, were recruited for study purposes. Subjects were excluded from the OAI if the following conditions were present: rheumatoid arthritis, bilateral knee arthroplasty or pre-existing plans to undergo bilateral (not unilateral) knee arthroplasty in the next 3 years, bilateral Osteoarthritis Research Society International (OARSI) stage 3 (severe) knee OA [12], positive pregnancy test, inability to provide a blood sample, use of ambulatory aids other than a single straight cane for >50% of the time, comorbid conditions that might interfere with 4-year participation, unlikely to reside in the clinic area for at least 3 years, current participation in a double-blind randomized controlled trial and being unwilling to sign an informed consent. Men above 130 kg and women above 114 kg were also excluded, due to specific requirements with the acquisition of magnetic resonance images, not relevant to the current study. In total, 27% (*n* = 4796) of all subjects screened (*n* = 17,457) were included in the study. The study was approved by the institutional review boards and was conducted in accordance with the Declaration of Helsinki. The clinical data are available for public use at https://nda.nih.gov/oai/. The study design protocol is available at https://oai.epi-ucsf.org/datarelease/docs/StudyDesignProtocol.pdf and has been previously described [14,22,23]. All variables used in this analysis were sampled from the publicly available OAI data base.

### 2.2. Sample Selection

Out of the 4796 OAI participants, 165 individuals were selected which received a femoro-tibial total knee replacement (TKR) during the interval between the baseline and the 4-year annual screening visit. The presence of a TKR was confirmed via radiographic assessment during a consecutive annual visit for each knee individually. Additionally, knees were classified as valid cases for the purposes of this study when a TKR was documented in the patient’s medical record.

Of those 165 individuals 24 (15%) received a TKR between the baseline and the 1-year screening visit, 33 (20%) between the 1-year and 2-year visit, 40 (24%) between 2-year and 3-year visit, 38 (23%) between 3-year and 4-year visit and 30 individuals (18%) between the 4-year and the 5-year screening visit. Detailed demographic data are shown in Table 1.

### 2.3. Clinical Data

Rearrangement of the data revealed that knee-specific clinical and imaging information was available for 140 knees 1 year before the TKR, for 107 knees 2 years before TKR, for 68 knees 3 years before TKR and for 30 knees information was available 4 years before TKR surgery (Table 2).

Radiographic classification of each knee relied on central radiographic readings (OAI release 0.5) assigning a Kellgren Lawrence grade (KLG) based on osteophytosis and joint space narrowing with higher grades indicating a worse knee state [24]. A knee showing evidence of a KL grade of more than II was classified as having radiographic evidence of knee osteoarthritis (ROA) [25,26].

The person specific quality of life was assessed via a subscore of the Knee Injury and Osteoarthritis Outcome Score (KOOS), ranging from 100–0, with higher values indicating a better quality [27].

The Western Ontario and McMaster Universities Osteoarthritis Index (WOMAC) total score and pain subscore was used to assess the functional and pain status of each individual knee and ranged from 0–100 or 0–20, respectively, with higher values indicating worse knee status [28].

Knee pain intensity was assessed via the numeric scale, ranging from 0 “no pain” to 10 “extreme pain” [29]. All variables included were publicly available and downloaded from the Osteoarthritis Initiative (OAI, version 3.2.1 clinical data): https://oai.nih.gov/.

Knees were classified as valid cases when a TKR was documented in the patient’s medical record and was additionally confirmed by radiography in the consecutive screening visits.

### 2.4. Analysis Strategy

Variables of interest were publicly available and sampled from the OAI database. Information obtained from the screening visits prior to the TKR surgery were re-arranged in order to be able to calculate differences between the years prior to the TKR surgery since the surgery could have occurred at any time point within the 4-year observational period. Differences in values were longitudinally compared between the years 0, 1, 2, 3, and 4 prior to TKR surgery. Variables were additionally included in the artificial neural networks in order to identify factors driving doctor’s decision for performing the surgical procedure in the OAI study population.

### 2.5. Statistical Methods

Data were analyzed for consistency, outliers and for normality using probability plots. Quality of life, WOMAC pain subscore, WOMAC total score and pain intensity were firstly tested globally by using Friedman’s ANOVA, and secondly, pairwise tests were performed by using Wilcoxon’s signed ranked test (based on the Monte Carlo method). 95% confidence intervals for medians and differences of medians were computed by using Hodge-Lehman intervals. KLG distributions were tested by using the marginal homogeneity test for dependent distributions and McNemar’s test.

Artificial neural networks (ANNs) with linear, radial basis function and three-layer perceptron neural networks architectures were used. A total of 14 variables were included into the model and the most predictive group consisting of pain intensity, Kellgren and Lawrence grades, WOMAC total score, used of medication (for pain, aching, or stiffness in the knee) and body mass index were identified by the integrated neural network variable selection algorithm to have the best outcome performance for predicting that a patient will undergo TKR within the next two years. For preprocessing, minimax coding and various activation functions were applied, i.e., linear, logistic and hyperbolic, whereas the linear function was used as a post synaptic potential function. More than 250 neural networks models with different network architecture were created and tested. Backpropagation method was used to train the network. In order to avoid overlearning, data were randomly split into a training, a verification and a test sample (ratio 2:1:1). A variable selection algorithm was used to find a subset of variables with low dimensions with sufficient prediction power. The process of identifying a proper network was monitored in the training, verification and test sample to avoid overlearning [30]. Negative and positive predictive value, sensitivity, specificity, percentage of unclassified and total percentage of correctly predicted patients were analyzed in each of these three samples. Instead of using one threshold, two prediction thresholds—one for acceptance and one for rejection—were used resulting into a grey area, i.e., if data of a subject fall into this area, no prediction is made. *p*-values less than 5% indicate a statistically significant difference. All analyses were done using PASW 22 (IBM SPSS Statistics for Windows, Version 19.0., Armonk, NY, USA), StatXact 10 (Cytel Software 2013, Cambridge MA, USA), Mathematica 7 (Wolfram Research, Inc., Mathematica, Version 7.0, Champaign, IL, USA), STATISTICA for neural networks 1.2 and STATISTICA 13 (Hill, T. & Lewicki, P. Statistics: Methods and Applications. StatSoft, Tulsa, OK, USA).

## 3. Results

### 3.1. Demographic Data

Demographic data of the 165 participants (60% females) included in this analysis revealed a mean age of 64.5 ± 8.4 years with a mean BMI of 29.7 ± 4.7 kg/m^2^. 85% of the study sample were of Caucasian ethnicity, 59% had an annual income of more than 50,000 USD and 50% had received during their education a college degree or above. Additional demographic data is shown in Table 1.

### 3.2. Change in Symptomology

**Figure 1:**
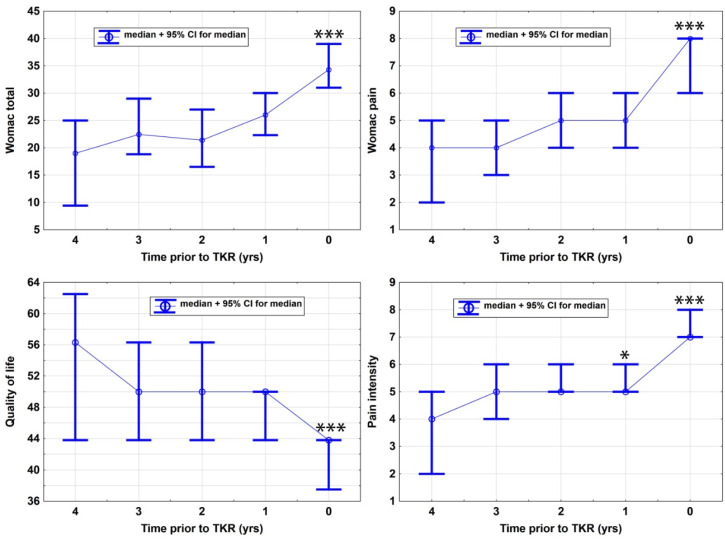
Panels show the longitudinal change of Western Ontario and McMaster Universities Osteoarthritis Index (WOMAC) total score [28], WOMAC pain subscore [28], pain intensity [29] and quality of life (as assessed by the knee specific Knee injury and Osteoarthritis Outcome Score (KOOS) [27]) in the years 4–0 prior to total knee replacement (TKR) (surgery. Significance levels are indicated by n.s. (not significant) or *: *p* ≤ 0.05, ***: *p* ≤ 0.001, and represent the comparison to the previous year: 3 vs. 4, 2 vs. 3, 1 vs. 2 and 0 vs. 1.; yrs: years.

Symptoms related to the knee affected by osteoarthritis increased sparsely in severity between 4 and 1 years prior to the TKR procedure for the WOMAC total score, the pain subscore, the knee related quality of life and knee pain intensity without reaching statistical significant worsening as compared between the annual screening visits year 4 to year 1 (Table 2 and Table 3). However, when compared between the years 1 and 0 prior to TKR surgery, symptom status worsened significantly in all of the measured variables: WOMAC total: 9.7 95% CI (7–12.5), *p* = < 0.0001; WOMAC pain subscore: 0.5 (1.5–3), *p* = < 0.0001; quality of life 9.4 (6.3–12.6), *p* = < 0.0001; and pain intensity 1.5 (1–2), *p* = < 0.0001 (Figure 1).

### 3.3. Structural Change as Evaluated by Kellgren and Lawrence Grading

**Figure 2:**
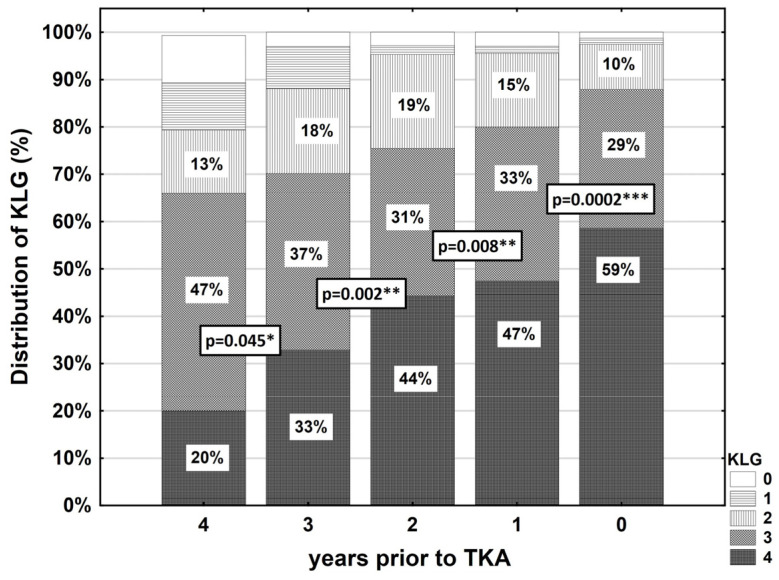
Longitudinal change in the distribution of Kellgren and Lawrence grades (KLG) ≥2 [19,20] of the total sample consisting of 165 individuals in the years 4–0 prior to total knee arthroplasty (TKA) surgery. Significance levels are indicated by *: *p* ≤ 0.05, **: *p* ≤ 0.01, ***: *p* ≤ 0.001, and represent the comparison to the previous year: 3 vs. 4, 2 vs. 3, 1 vs. 2 and 0 vs. 1. Statistical testing was performed with McNemar’s test.

Four years prior TKR, 80% of the sample was graded as KL grade 2 or worse, 88% as KLG > 1 three years prior TKR, 96% as KLG > 1 three years prior TKR and 98% in the year prior to the TKR procedure (Table 2). There was a significant worsening in KL grades between each of the annual screening visits: 4–3 years: *p* = 0.045, 3–2 years: *p* = 0.002, 2–1 years: *p* = 0.008 and in the year prior TKR i.e., 1–0 years: *p* = 0.0002 (Figure 2 and Table 3).

### 3.4. Prediction of TKR Using Artificial Neural Networks

**Figure 3:**
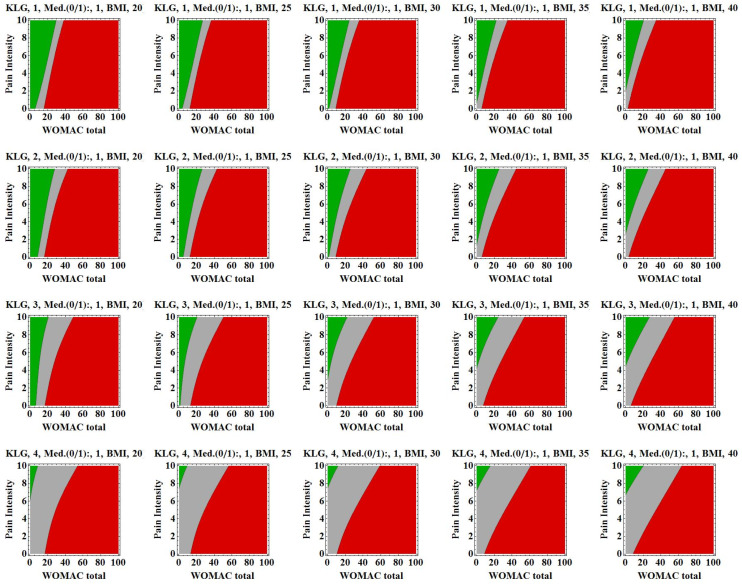
Graphic visualization of the neural network output illustrating the three-layer perceptron decision areas including the variables pain intensity, Kellgren and Lawrence grades, Western Ontario and McMaster Universities Osteoarthritis Index (WOMAC) total score, use of medication (Med.; 0 = taking no pain medication, 1 = taking pain medication) and body mass index into the neural network. Areas represent: green area = no TKR within next 2 years, red area = TKR within next 2 years and grey area = no prediction is made. The following scenario can be hypothesized: for a patient with a Kellgren and Lawrence grade of 1, a body mass index of 25 kg/m^2^, a WOMAC total score of 20 and a pain intensity of 8, it is predicted that TKR surgery is not appropriate within the next 2 years, according to previous decisions taken in the Osteoarthritis Initiative (OAI) population. BMI: Body Mass Index.

After applying the variable selection algorithm and testing more than 250 models, KL grades, WOMAC total score, body mass index measures, pain intensity and pain medication revealed the following performance: negative predictive value 73% (52–88%), positive predictive value 84% (71–94%), specificity 30% (19–42%), sensitivity 41% (27–57%), percentage of unclassified knees 54% (46–62%) and total percentage of correctly predicted knees 80% (69–89%) (Figure 3).

## 4. Discussion

Our data revealed that structural change as evaluated by the x-ray based Kellgren and Lawrence grading a significant worsening occurred between every of the four annual visits prior to the TKR procedure. When analyzing the symptomology of the investigated 165 included participants, as assessed by the WOMAC total score, by the WOMAC pain subscore, by the knee specific quality of life or by the knee pain intensity, a significant worsening was detected only in the year prior to the TKR procedure but in not in the years earlier. When including selected factors into our artificial neural network model, we were able to compute that 80% of all patients with a prediction were correctly predicted with a positive predictive value of 84% and a negative predictive value of 73% for the total model.

One limitation of this study is that we investigated a subpopulation of the OAI, which had specific criteria for including participants into the study that had present or being at risk of developing symptomatic knee osteoarthritis [10,11]. This might not reflect the general population, as knee OA subtypes such as post-traumatic knee OA or individuals at a young age (<45 years) were not considered specifically. On the other hand, the OAI provides the unique opportunity for researchers to sample publicly available datasets with having a specific population that is prone to receiving a TKR within the duration of the study. This would be less feasible in other experimental settings that emphasize the uniqueness of the data used in the present study to obtain representative results in a rationale longitudinal follow-up. Another limitation of the present study is that the outcome of the TKR procedure and the long-term outcome were not considered in our prediction model as previously performed in study by Escobar et al. [14]. In this study, the appropriateness for the indication to TKR surgery in patients with osteoarthritis was re-evaluated also in consideration of the outcome of the surgical procedure. This allows one to include the effects of the taken decision into the decision-making process, but it is also prone to blurring the integrated factors due a good outcome independent of the factors included into the model. Another limitation of this study is that the patients included in this study were treated at different institutions and the decision to perform TKR surgery is an individual one and can vary between centers and even between doctors within one center.

It is widely accepted that radiographic assessments of the knee serve as a good parameter when trying to evaluate a patients’ need for TKR [8,9,10,11,12] and that radiographic end-stage knee OA is the key-factor for taking the decision to perform KKR surgery [6,7]. A limitation to this objective parameter is that a patients’ symptom status, i.e., pain, disability and quality of life, are not reflected. Our results confirm this observation, as 98% of the individuals have definite radiographic knee OA with a Kellgern and Lawrence score of 2 or worse. However, four individuals had a KL score of 0 or 1 and were nevertheless scheduled for TKR surgery. This interesting fact receives support by our observations on the symptomology in the years prior to the procedure. In the years prior to TKR the worsening between the annuals screening visits was not significant as compared to the preceding screening visit in the years four to one prior to TKR. However, in the year prior to TKR, the symptoms, as assessed by the WOMAC total and pain subscore, by the KOOS quality of life or by knee pain intensity, worsened significantly as compared to the year prior to TKR. These factors can be regarded as the major driving factors for the decision making, as during the following year, TKR surgery was performed in these individuals, independent of their radiographic knee status. 

In order to have the possibility to predict other patients’ need for TKR surgery, i.e., other than those included in the OAI, we performed analyses using artificial neural networks. Artificial neural networks support the decision making process based on input variables for a defined outcome. As all of the individuals included in our investigation received a TKR during the observational period as a determined outcome, we included 14 variables representing information on structural, symptomatic, socio-economic and demographic influencing factors. The model used a selection algorithm to select six variables that had, in combination, the best performance. As in general such models perform great in a given sample, it has been shown that they fail when they are applied to predict new cases.

Yu et al. established two prediction models for TKR and total hip replacement using Cox proportional hazards models based on two large cohorts [31]. This study yielded well-performing risk prediction equations however they established their model based on epidemiological patient data, whereas we implemented both epidemiological data as well as data on knee functionality, symptomology and radiographical status of the patients [31]. Furthermore, our prediction model is easily applicable using the figure presented in this study. Another study conducted by Zeni et al. used a logistic regression model to identify predictive variables however the patient specific data was not analyzed longitudinally in the course of time prior to TKR and no prediction model was established [20]. Nevertheless, the study by Zeni et al. also emphasizes the crucial role of knee functionality in regard to the risk of TKR [20]. Additionally, various studies have shown the relevance of magnetic resonance imaging (MRI) in regard to TKR prediction but MRI is still not as easily accessible as conventional radiographs [16,17]. Gossec et al. have previously shown that radiological grade, mean patient global assessment and the need for NSAIDs were predictive of total hip replacement, which corresponds well to our results for TKR [18]. Chan et al. created a formula that provides acceptable diagnostic efficacy in retrospect for the prediction of TKR based on similar variables as our prediction model, which suggests that our model focuses on appropriate predictive factors [19]. Concludingly our study takes a broad variety of variables into account, while the prediction model remains easily applicable in a primary care setting.

The uniqueness of our present model is that we used a subsample of our total sample, i.e., out of 165 individuals to train the model, another subsample to verify the training effect and another subsample (not included in the model thus far) to calculate the performance of the applied artificial neural networks. As a result, of those who were able to be classified, 80% were classified correctly. Another unique fact of our applied model is that we used two thresholds, i.e., one for acceptance and one for rejection with leaving a “grey area,” in which a classification, i.e., a TKR procedure that will be performed within the next 2 years, was not possible.

As stated previously by Fei et al., artificial neural networks (ANN) have exceptional information processing abilities learning and generalization capabilities, and fault and noise tolerance; moreover, ANNs were able to assess more variables than logistic regression [32,33,34]. In brief, ANNs can be seen as enhancements of classic regression models [30,35]. As reviewed by Miller et al. ANNs have been successfully applied in terms of diagnostic applications and are likely to play an important role in regard to diagnostics and treatment of chronic diseases in the future [36]. Taking these advantages into account, we decided to establish our prediction model based on ANNs. However, this specific technique is not able to analyze and evaluate individual factors, while logistic regression modelling can provide this kind of information, which can be seen as a limitation of our prediction model [32]. Another limitation of our study and ANNs in general are the “black-box” characteristics of this technique, making it extremely difficult to determine how certain processes are performed [37]. Another limitation to our study is the relatively small sample size; therefore, further studies will be needed to improve and validate our prediction model. However, we believe that our novel approach using ANNs to predict the risk of TKR is of clinical value, and the applied technique is well-suited to address this controversially discussed topic.

The mathematical basis of our model is provided in the Supplementary Material: The Mathematical Basis of this paper and can be easily implemented in an Excel worksheet for the daily use in clinical care.

## 5. Conclusions

Longitudinal analyses of symptomology and structural change in a four-year period prior to TKR surgery revealed that the significant worsening in pain, function and quality of life in the year prior to the TKR procedures seems to be the more dominant decision driving factor as compared to the radiographic structural progression of the knee. Analyzing a total of 14 different structural, symptomatic, socio-economic and demographic factors via artificial neural networks, we were able to establish a prediction model that predicted correctly 80% of the classified individuals to undergo TKR surgery within the next 2 years with a positive predictive value of 84% and a negative predictive value of 73%. Considering that our prediction model is based on easily accessible patient data, it could be used in a primary care setting to evaluate the need for TKR surgery within the next two years.

## Figures and Tables

**Table 1:** Baseline demographic data of the 165 participants included in this investigation. Information not known or unwilling to give by the participants during the screen visits were classified as not available. Values are given as quantity with the respective percentage of the total sample (%) or as means with standard deviation (SD).

**Demographics**	
Females (%)	100 (60)
Age (SD)	64.5 (8.4)
Body Mass Index (SD)	29.7 (4.7)
**Ethnicity**	
Other Non-White (%)	2 (1.2)
Caucasian (%)	140 (85)
African American (%)	20 (12)
Asian (%)	2 (1.2)
Not Available (%)	1 (0.6)
**Medication**	
Any Medication Used for Pain, Aching or Stiffness (%)	98 (59)
**Annual Income in United States Dollar (USD)**	
Less Than 10,000 (%)	2 (1.3)
10,000 < 25,000 (%)	16 (9.7)
25,000 < 50,000 (%)	41 (25)
50,000 < 100,000 (%)	57 (35)
>100,000 (%)	39 (24)
Not available (%)	10 (6)
**Education**	
Less Than High School (%)	6 (3.5)
High School (%)	31 (19)
Any College (%)	44 (27)
College Graduate (%)	29 (18)
Any Graduate School (%)	12 (7)
Graduate Degree (%)	42 (25)
Not Available (%)	1 (0.5)
**Depression**	
At Risk for Clinical Depression (%)	15 (9)
**Start of Knee Symptoms Prior to Baseline Screening Visit**	
None (%)	11 (6.6)
0–1 year (%)	21 (12.6)
2–5 years (%)	47 (28.1)
> 5 years (%)	67 (40.1)
Not Available (%)	21 (12.6)
**Total Knee Replacement at Which Year**	
Between Baseline and 1-Year Follow-up Screening Visit (%)	24 (15)
Between 1-Year and 2-Year Follow-up Screening Visit (%)	33 (20)
Between 2-Year and 3-Year Follow-up Screening Visit (%)	40 (24)
Between 3-Year and 4-Year Follow-up Screening Visit (%)	38 (23)
Between 4-Year and 5-Year Follow-up Screening Visit (%)	30 (18)

Ethnicity: distribution of ethnical groups among patients; Medication: proportion of patients taking any medication used for pain, aching or stiffness at baseline screening visit; Annual Income In United States Dollar (USD): distribution of annual income among patients at baseline screening visit; Education: distribution of highest educational levels among patients at baseline screening visit; Depression: proportion of patients who are at risk for clinical depression at baseline screening visit; Start of Knee Symptoms Prior to Baseline Screening Visit: distribution of duration of knee symptoms prior to baseline screening visit among patients; Total Knee Replacement at Which Year: distribution of timepoints at which patients received total knee replacement.

**Table 2:** Descriptive data of the 165 study participants at the following time points prior to the total knee replacement (TKR) surgery: 0 years (last follow-up visit without TKR i.e., in the next annual follow-up visit radiographic evidence for a total knee replacement was detected), 1 year (second last visit without TKR i.e., in the annual follow-up visit in 2 years radiographic evidence for a total knee replacement was detected), 2 years (third last visit without TKR i.e., in the annual follow-up visit in 3 years radiographic evidence for a total knee replacement was detected), 3 years (fourth last visit without TKR i.e., in the annual follow-up visit in 4 years radiographic evidence for a total knee replacement was detected) and 4 years (fifth last visit without TKR i.e., in the annual follow-up visit in 5 years radiographic evidence for a total knee replacement was detected) prior to TKR. Quality of life was measured by a subscale of the Knee injury and Osteoarthritis Outcome Score (KOOS) 0–100 (worst to best). Functional measures were evaluated by the Western Ontario and McMaster Universities Osteoarthritis Index (WOMAC) Total score 0–100 (best to worst), whereas pain measures relied on the WOMAC pain subscale 0–20 (best to worst). Pain intensity was captured by the numerical rating scale estimating the participant’s pain intensity during the last 30 days 0–10 (best to worst). Values are given as median with the accompanying 25% and 75% quartiles or as count and percent (%) for the Kellgren and Lawrence grades.

	0 Years Prior TKR	1 Year Prior TKR	2 Years Prior TKR	3 Years Prior TKR	4 Years Prior TKR
	*n* = 165	*n* = 140	*n* = 107	*n* =68	*n* =30
Quality of Life	43 (25–56.3)	50 (37.5–62.5)	50 (31.3–62.5)	50 (37.5–62.5)	56.3 (43.8–68.8)
WOMAC Pain Subscore	8 (4–11)	5 (3–8)	5 (2–7.25)	4 (3–7)	4 (0–6)
WOMAC Total Score	34.3 (24–48)	26 (12–36.1)	21.4 (9.2–37.5)	22.5 (10.4–34.7)	19 (4–27)
Pain Intensity	7 (5–8)	5 (4–7)	5 (4–7)	5 (3–6)	4 (2–6)
Kellgren and Lawrence Grades (%) ^1^	***	**	**	*	
0	2 (1)	4 (3)	3 (3)	2 (3)	3 (10)
1	2 (1)	2 (1)	2 (2)	6 (9)	3 (10)
2	15 (9)	21 (15)	21 (19)	12 (18)	4 (13)
3	46 (28)	44 (31)	33 (31)	25 (37)	14 (47)
4	92 (56)	64 (46)	47 (44)	22 (32)	6 (20)
Not Available	8 (5)	5 (4)	1 (1)	1 (1)	0 (0)

^1^ Significant difference were calculated by comparison to the previous years’ visit using a marginal homogeneity test. Significances were: *** *p* < 0.001; ** 0.001 < *p* <0.01; * 0.01 < *p* < 0.05.

**Table 3:** Longitudinal comparative data of the 165 study participants at the following time points prior to the total knee replacement (TKR) surgery: 0 years (last follow-up visit without TKR i.e., in the next annual follow-up visit radiographic evidence for a total knee replacement was detected), 1 year (second last visit without TKR, i.e., in the annual follow-up visit, in 2 years, radiographic evidence for a total knee replacement was detected), 2 years (third last visit without TKR, i.e., in the annual follow-up visit, in 3 years, radiographic evidence for a total knee replacement was detected), 3 years (fourth last visit without TKR, i.e., in the annual follow-up visit, in 4 years, radiographic evidence for a total knee replacement was detected) and 4 years (fifth last visit without TKR, i.e., in the annual follow-up visit, in 5 years, radiographic evidence for a total knee replacement was detected) prior to TKR. Quality of life was measured by a subscale of the Knee injury and Osteoarthritis Outcome Score (KOOS) 0–100 (worst to best). Functional measures were evaluated by the Western Ontario and McMaster Universities Osteoarthritis Index (WOMAC) Total score 0–100 (best to worst), whereas pain measures relied on the WOMAC pain subscale 0–20 (best to worst). Pain intensity was captured by the numerical rating scale estimating the participant’s pain intensity during the last 30 days 0–10 (best to worst). Values are given as median with the accompanying 25% and 75% quartiles. For comparisons between change in Kellgren and Lawrence grades (KLG) we compared the strata having KLG 0 and 1 versus KLG 2, 3 and 4 (also see Figure 2).

	0 vs. 1 Years	1 vs. 2 Years	2 vs. 3 Years	3 vs. 4 Years
	Median Difference, 95% CI, *p*-value			
Quality of Life ^1^	9.4 (6.3–12.6) *p* < 0.0001 ***	0 (−3.2–3.2) *p* = 0.73	0 (−6.2–6.2) *p* = 0.99	0 (−6.2–12.5) *p* = 0.32
WOMAC Pain Subscore ^1^	0.5 (1.5–3) *p* < 0.0001 ***	0.5 (−0.3–1) *p* = 0.27	0 (−1.0–1.0) *p* = 0.75	1.2 (−0.5–3) *p* = 0.12
WOMAC Total Score ^1^	9.7 (7–12.5) *p* < 0.0001 ***	2.6 (−0.1–5.4) *p* = 0.062	1.8 (−1.5–4.9) *p* = 0.24	2.3 (−1.8–7.2) *p* = 0.23
Pain Intensity ^1^	1.5 (1–2) *p* < 0.0001 ***	0.5 (0.3–1.5) *p* = 0.014 *	0 (−0.5–1.0) *p* = 0.89	0 (−1.0–1.5) *p* = 0.92
Change in KLG ≥ 2				
	0 vs. 1 years	1 vs. 2 years	2 vs. 3 years	3 vs. 4 years
Kellgren and Lawrence grades ^2^	*p* = 0.0002 ***	*p* = 0.008 *	*p* = 0.002 *	*p* = 0.045 *

^1^ Significant differences were calculated by comparison to the previous years’ visit using a global Friedman test and local using Wilcoxon signed ranked test based on Monte Carlo methods and ^2^ McNemar Test (one sided). Significances were: *** *p* < 0.001; * 0.01< *p* < 0.05.

## Supplementary Materials

The following are available online at https://www.mdpi.com/2077-0383/9/5/1298/s1, The Mathematical Basis.
