# Supplementary material for: Predicting Total Knee Replacement from Symptomology and Radiographic Structural Change Using Artificial Neural Networks—Data from the Osteoarthritis Initiative (OAI)
Source: J Clin Med. 2020 May 1;9(5):1298. doi: 10.3390/jcm9051298 (PMC7288322; doi:10.3390/jcm9051298)
Supplement: Supplementary file 1 [file jcm-09-01298-s001.pdf]

Supplementary material: The Mathematical Basis

Body mass index is ranging from 21–44, KL grades from 1-4, medication is coded as 1 if any, used for pain, aching or stiffness and 0 otherwise. Womac total is ranging from 0 to 70 and pain intensity from 0 to 10.

1. Let X_1 be the input vector with X_1=(BMI, KLG, medication, womac total, pain intensity) and $g:=(g_1,g_2,g_3,g_4,g_5)$ be the minimax transformation with $g_1(X_1):=-0.942+0.04464$ BMI, $g_2(X_1):=-0.333+0.333$ KLG, $g_3(X_1)$:=medication, $g_4(X_1):=0.01447$ womac total and $g_5(X_1):=0.1$ pain intensity. Let $X_2:=(-1,g(X_1))$, h be the activation function with $h(x):=(1+\mathrm{Exp}(-x))^{-1}$ and W_1 the weight matrix

$$W_1 = \begin{pmatrix} -1.1497 & -1.16983 & 0.714297 & -0.2071 & -0.1417 \\ -0.1486 & -0.269 & 3.01519 & 0.09788 & 1.41995 \\ 3.83908 & 0.433202 & 3.44988 & 1.05708 & -1.48424 \\ -1.23185 & -0.3312 & -1.62882 & 0.447729 & -1.51218 \\ -5.27405 & 0.07767 & 5.58068 & 2.32511 & -2.34568 \\ 3.22912 & 0.639357 & 1.03647 & -0.3097 & 3.2952 \end{pmatrix}$$

We define $X_3:= W_1^T.X_2$ and $X_4:=(h(X_{3,1}), h(X_{3,2}), h(X_{3,3}), h(X_{3,4}), h(X_{3,5})$. The second weight matrix is given by

$$W_2 = (-0.1118 \quad -5.703198 \quad -0.9107 \quad 5.097852 \quad 2.661026 \quad -2.440197)$$

Now let $X_4:=W_2.(-1,X_4)$ and $X_5:=(h(X_{4,1}), h(X_{4,2}), h(X_{4,3}), h(X_{4,4}), h(X_{4,5}))$.
The artificial neural network uses the following decision rule: if $X_4 < 0.35$, then TKR ≤ 2 years is predicted, if $0.35 \leq X_4 \leq 0.75$, then no prediction is made and if $0.75 < X_4$, then TKR > 2 years is predicted.
